# Additive diagnostic value of thoracic SPECT/CT imaging in perugini grade 1 patients who underwent bone scintigraphy

**DOI:** 10.1007/s00259-025-07653-w

**Published:** 2025-11-12

**Authors:** Michael Poledniczek, René Rettl, Christina Kronberger, Lena Marie Schmid, Nikita Ermolaev, Franz Duca, Christian Nitsche, Christina Binder, Luciana Camuz Ligios, Mahshid Eslami, Patrick Binder, Clemens P. Spielvogel, Roza Badr Eslam, Dietrich Beitzke, Johannes Kastner, Jutta Bergler-Klein, Andreas A. Kammerlander, Christian Hengstenberg, Marcus Hacker, Raffaella Calabretta

**Affiliations:** 1 Division of Cardiology, Department of Internal Medicine II, Medical University of Vienna, Vienna, Austria; 2 Division of Nuclear Medicine, Department of Biomedical Imaging and Image-Guided Therapy, Medical University of Vienna, Waehringer Guertel 18-20, Vienna, 1090 Austria; 3 Division of Cardiovascular and Interventional Radiology, Department of Biomedical Imaging and Image-Guided Therapy, Medical University of Vienna, Vienna, Austria

**Keywords:** Cardiac amyloidosis, Scintigraphy, SPECT/CT, [^99m^Tc]-DPD, Left ventricular uptake quantification

## Abstract

**Purpose:**

Left ventricular (LV) myocardial uptake of ^99m^Technetium-labeled tracers is assessed to diagnose transthyretin amyloid cardiomyopathy (ATTR-CM). The degree of uptake is visually graded using planar images utilising the Perugini score. Today, non-invasive diagnosis of ATTR-CM is broadly established in practice; however, in patients with mild tracer uptake (Perugini grade 1), no definite diagnosis can be made without endomyocardial biopsy.

**Methods:**

Within the scope of a prospective cardiac amyloidosis registry at the Medical University of Vienna, all patients who underwent bone scintigraphy graded as Perugini grade 1 with additional SPECT/CT imaging performed between September 2014 and May 2025 were retrospectively analysed.

**Results:**

41 patients (70.8 years, IQR: 64.9–78.8, 41.5% female) were included. The majority (*n* = 32, 78.0%) of scans were ordered for a suspicion of ATTR-CM. On SPECT/CT images, true LV tracer uptake was confirmed in 4 (9.8%) patients, and 1 (2.4%) patient presented with focal myocardial uptake. In all other patients, tracer uptake was not within the myocardial tissue but rather blood-pool uptake. In follow-up [^99m^Tc]-DPD scintigraphy, myocardial tracer uptake eventually progressed to Perugini grade 2 in 3 patients who previously demonstrated mild LV myocardial tracer uptake. In contrast, those with diffuse LV uptake did not show any signs of progression in follow-up SPECT/CT imaging.

**Conclusion:**

SPECT/CT is mandatory in patients with mild mediastinal tracer uptake interpreted as Perugini grade 1. Among patients with Perugini grade 1 and confirmed [^99m^Tc]-DPD LV uptake on SPECT/CT images, progression to Perugini grade 2 was observed in all individuals who underwent nuclear medicine imaging follow-up.

## Introduction

Transthyretin amyloid cardiomyopathy (ATTR-CM) is a progressive infiltrative disease of the myocardium [1]. The clinical presentation is heterogenous and resembles various aetiologies of heart failure with preserved ejection fraction (HFpEF) [2, 3]. While ATTR-CM is considered to be among the most malignant causes of HF, patients often go undiagnosed for too long [4, 5].

In 2016, Gillmore et al. published a non-invasive diagnostic algorithm for individuals with suspected cardiac amyloidosis, which combines bone scintigraphy with electrophoresis and immunofixation of serum and urine (IFIX) and free light chain (FLC) assessment [6]. Myocardial uptake of [^99m^Technetium]- ([^99m^Tc])-labelled bone-seeking tracers is assessed and rated visually on scintigraphy images according to a score established by Perugini et al. [7]. In patients with an unremarkable IFIX and FLC result, Perugini grades 2 or 3, i.e., moderate or strong cardiac tracer uptake, are considered diagnostic for ATTR-CM [7]. Today, non-invasive diagnosis of ATTR-CM is broadly established in practice [6, 8]. The excellent positive predictive value of this constellation of non-invasive diagnostic workup has been re-evaluated in several studies and confirmed by a meta-analysis [9]. However, in patients whose IFIX or FLC assessments demonstrate abnormal results, caution is warranted. In these patients, endomyocardial biopsy (EMB) is still an indispensable diagnostic procedure for the evaluation of suspected cardiac amyloidosis.

In clinical practice, some patients present with a Perugini grade 1 (suggestive of mild myocardial tracer uptake) on whole-body bone scintigraphy and an unremarkable IFIX/FLC. In these patients, a non-invasive diagnosis is impossible, and EMB or extracardiac biopsy in conjunction with cardiac magnetic resonance (CMR) or transthoracic echocardiography (TTE) findings in line with cardiac amyloidosis are required [10]. Especially in patients with low-grade cardiac tracer uptake, additional thoracic single photon-emission computed tomography/low-dose computed tomography (SPECT/CT) imaging is considered valuable in discerning false positives, which might occur due to blood pool uptake, hydroxychloroquine cardiac toxicity, valvular/annular uptake, or recent myocardial infarction [10]. We therefore aimed to retrospectively analyse the additional diagnostic value of thoracic SPECT/CT images over planar bone scintigraphy with [^99m^Tc]−3,3-diphosphono-1,2-propanodicarboxylicacid ([^99m^Tc]-DPD) in Perugini grade 1 patients and their final diagnoses in our single-centre cohort. Therefore, we aim to gain a better understanding of mild left ventricular (LV) myocardial tracer uptake to improve patient selection for an invasive diagnostic tool such as EMB.

## Materials and methods

### Setting

**Fig. 1:**
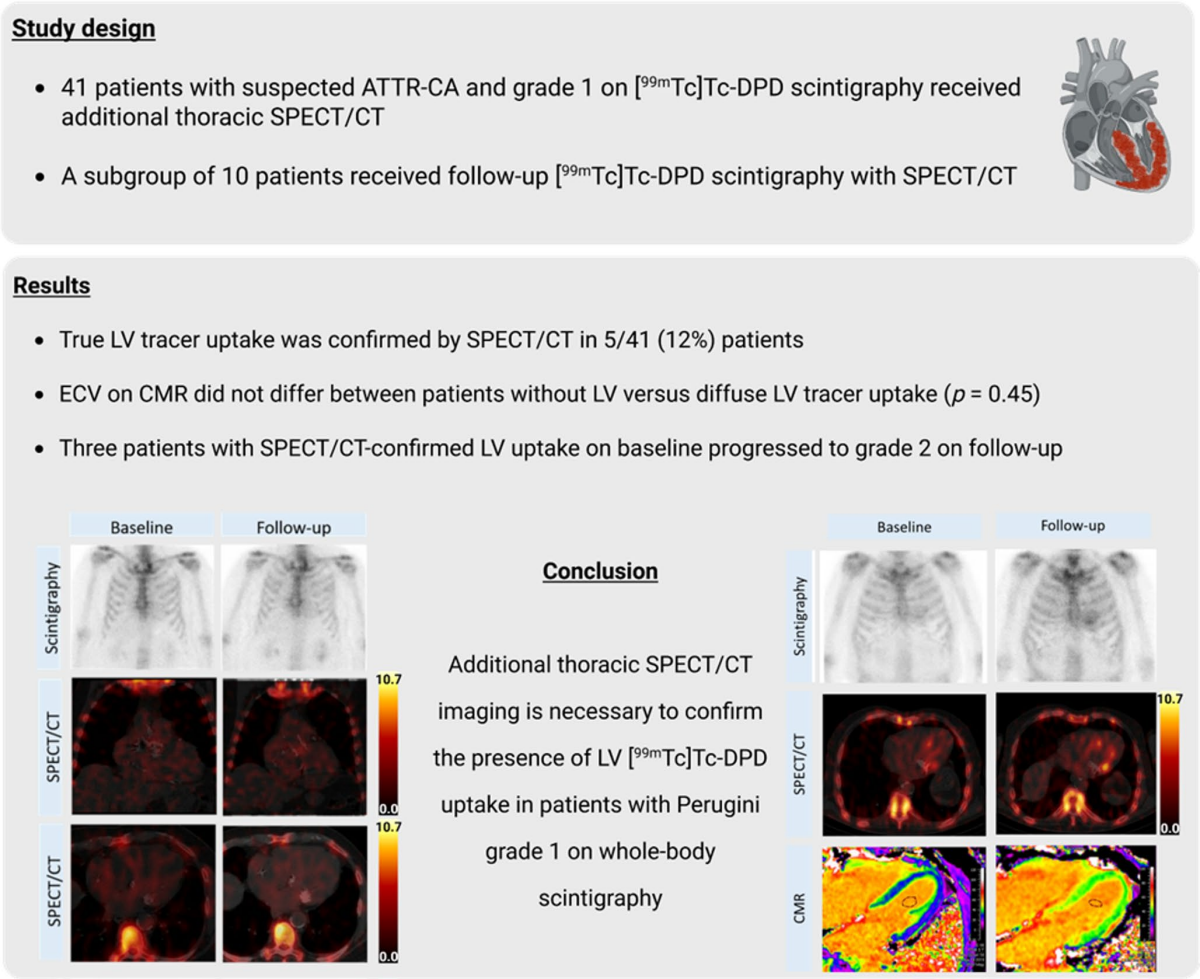
Graphical abstract

This analysis was conducted within the scope of a prospective cardiac amyloidosis registry at the tertiary referral centre for cardiac amyloidosis at the Medical University of Vienna, Department of Medicine II, Division of Cardiology, Vienna, Austria. In addition, all [^99m^Tc]-DPD bone scintigraphy examinations graded as Perugini grade 1 with additional thoracic SPECT/CT imaging performed between September 2014 and May 2025 were retrospectively analysed. The study was approved by the Ethics Committee of the Medical University of Vienna (EK 1019/2023 and 2352/2024, respectively) and was carried out in accordance with the Declaration of Helsinki. Participants provided written informed consent before enrolment in the registry, and informed consent was waived for retrospectively included patients who did not present to the cardiac amyloidosis unit. A graphical overview of the study is shown in the Fig. 1.

### Study population and design

All patients underwent [^99m^Tc]-DPD bone scintigraphy and additionally quantitative SPECT/CT of the thorax at the Department of Biomedical Imaging and Image-guided Therapy, Division of Nuclear Medicine at the Medical University of Vienna. Patients were included with evidence of Perugini grade 1 on planar bone scintigraphy. Patients were excluded with Perugini grade 2 or 3 as well as in the presence of abnormal results in IFIX and FLC. If patients underwent repeated [^99m^Tc]-DPD scintigraphies, subsequent examinations were also included. A diagnosis of ATTR-CM was considered established if the non-invasive diagnostic criteria [6] were met, or through EMB and subsequent Congo-red staining.

### Nuclear medicine imaging

[^99m^Tc]-DPD bone scintigraphy was performed using a dedicated SPECT/CT system (Symbia Intevo, Siemens Medical Solutions AG, Erlangen, Germany). Patients received approximately 740 MBq of the radionuclide tracer [^99m^Tc]-DPD intravenously. After approximately three hours, whole-body scintigraphy was acquired at a scan speed of 10 cm/min using low-energy high-resolution collimators immediately followed by a quantitative SPECT/CT of the chest. No specific preparation was required, aside from bladder voiding immediately before imaging. Whole-body scans were conducted using low-energy high-resolution collimators in a 180° configuration, with a scanning speed of 25 cm/min, matrix size of 256 × 1024, and an energy window of 15% centred around the 140.5 keV photopeak of [^99m^Tc]Tc.

SPECT imaging was also performed in a 180° configuration using step-and-shoot acquisition with body contouring. A total of 64 projections were acquired, each with a duration of 20 s, using a 256 × 256 matrix. An energy window of 15% around the 140.5 keV photopeak of [^99m^Tc]Tc and a 15% lower scatter window were applied. CT scans for attenuation correction were performed as low-dose acquisitions (130 kV, 35 mAs, 512 × 512 matrix). SPECT/CT images were reconstructed using the xSPECT/CT-QUANT iterative algorithm (256 × 256 matrix), incorporating attenuation correction. Cross-calibration was performed using a local dose calibrator (Isomed 2010) and a [^99m^Tc]Tc point source. An experienced nuclear medicine physician (R.C.) using advanced imaging software provided by Hermes Medical Solutions (Stockholm, Sweden) performed the image analysis and interpretation.

**Fig. 2:**
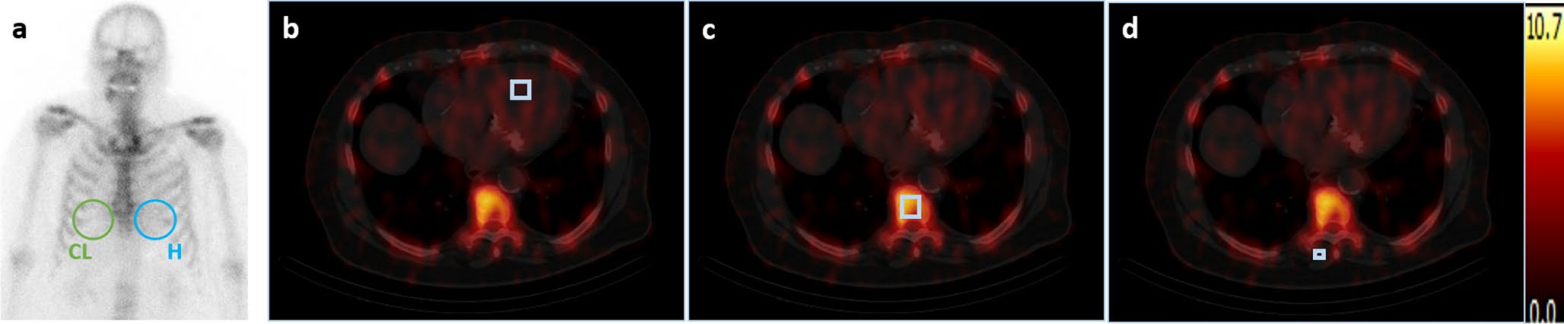
Nuclear medicine imaging analysis. Whole-body planar images were analysed semi-quantitatively by deriving the H/CL ratios (**a**). LV myocardial [^99m^Tc]-DPD-uptake was quantified on the SPECT/CT images as peak standardised uptake value (SUV_peak_) by placing manually cubic VOIs within the myocardial tissue of the interventricular septum (2 ml) (**b**), on the 8th thoracic vertebra (2.92 ml) (**c**) as well as on the left paraspinal muscle (1.19 ml) (**d**), respectively. The tracer retention index was additionally derived. [^99m^Tc]-DPD, [^99m^Tc]−3,3-diphosphono-1,2-propanodicarboxylicacid; H/CL, heart-to-contralateral; SPECT/CT, single photon emission computed tomography/computed tomography; VOIs, volumes of interest

Whole-body images were analysed visually, by using the Perugini score, and semi-quantitatively, by deriving the heart-to-contralateral (H/CL) ratios (Fig. 2a), as previously described [11]. LV myocardial [^99m^Tc]-DPD-uptake was quantified on the SPECT/CT images as peak standardised uptake value (SUV_peak_) and the tracer retention index (RI) was derived, as previously explained [12]. Volumes of interest (VOIs) were placed manually within the myocardial tissue of the interventricular septum (2 ml), on the 8th thoracic vertebra (2.92 ml) as well as on the left paraspinal muscle (1.19 ml), respectively (Fig. 2b-d).

### Further assessments and evaluations

Patients within the cardiac amyloidosis registry were evaluated with regard to current symptoms, medical history and concomitant medication. Standard laboratory measurements included a complete blood count, blood chemistry and coagulation panel. In addition, biomarkers of heart failure, i.e., N-terminal prohormone of brain natriuretic peptide (NT-proBNP) and troponin t were obtained. Genetic testing for variant transthyretin amyloidosis (ATTRv) was offered to all patients who received a diagnosis of ATTR-CM.

CMR was routinely performed on a 1.5 Tesla cardiac-dedicated clinical magnetic resonance system (Avanto Fit, Siemens Medical Solutions, Erlangen, Germany). The CMR protocol consists of a functional study and late gadolinium enhancement (LGE) imaging. Cardiac function, myocardial mass and LGE were analysed and indexed to body surface area. Parametric mapping (T1-mapping, T2-mapping, ECV) is performed as previously described [13].

In retrospectively included patients, electronic hospital documentation was searched for clinical characteristics, laboratory assessments and final diagnosis.

### Diagnosis and follow-up

The final diagnosis of patients was retrieved either by review of in-hospital or out-of-hospital electronic patient documentation or telephone interview. In patients with persistent suspicion of cardiac amyloidosis, [^99m^Tc]-DPD scintigraphy and SPECT/CT imaging were repeated after a median of 26 (17–49) months to evaluate possible temporal changes of myocardial tracer uptake.

### Statistical analysis

Statistical analysis was performed using BlueSky Statistics 10.3.4, R package version 8.95 (BlueSky Statistics LLC, Chicago, IL, USA). Continuous variables are expressed as median and interquartile ranges (IQR) or mean and standard deviation depending on the respective variables’ distribution, assessed using the Shapiro-Wilk test. Categorical variables are presented as numbers and percentages. Baseline and follow-up variables were compared using the Wilcoxon signed-rank test and McNemar’s test as appropriate. Statistical significance was assumed with p-values from two-sided tests of < 0.05.

## Results

### Baseline characteristics

A total of 41 patients (70.8 years, IQR: 64.9–78.8, 41.5% female) with Perugini grade 1 and unremarkable results in IFIX/FLC constituted the final study population. The baseline characteristics of the entire study population are depicted in Table 1. Patients presented with elevated biomarkers of heart failure (NT-proBNP: 1126 pg/ml, IQR: 422–1919) and reduced kidney function (eGFR: 56.1 ± 24.2 ml/min/1.73m^2^). Most of the patients analysed were in New York Heart Association (NYHA) functional class II (*n* = 14, 56.0%). The most prevalent comorbidity was arterial hypertension (*n* = 27, 65.9%), followed by chronic kidney disease (*n* = 24, 58.5%) and atrial fibrillation (*n* = 20, 48.8%). A history of malignancy was recorded in 14 (34.1%) patients.

**Table 1:** Baseline characteristics of the patient cohort

Variable	Total	Myocardial uptake	Diffuse uptake	*P*-value
	*N* = 41	*N* = 5	*N* = 36	
**Demographics**				
Age (years), median (IQR)	70.8 (64.9–78.8)	79.1 (76.2–80.2)	69.3 (64.2–77.9)	*0.135*
Female sex, n (%)	17 (41.5%)	1 (20.0%)	16 (44.4%)	*0.299*
**Pre-test probability & red flags**				
History of carpal tunnel syndrome, n (%)	7 (20.0%)	3 (60.0%)	4 (13.3%)	***0.016***
History of spinal canal stenosis, n (%)	3 (8.6%)	1 (20.0%)	2 (6.7%)	*0.324*
Apical sparing pattern (%)	2 (6.9%)	2 (40.0%)	0 (0.0%)	***0.001***
**Clinical characteristics**				
Height, cm, mean ± SD	170 ± 9.6	173.6 ± 8.1	169.4 ± 9.7	*0.363*
Weight, kg, mean ± SD	82.5 ± 14.9	77.8 ± 6.8	83.1 ± 15.7	*0.463*
BMI, kg/m^2^, mean ± SD	28.6 ± 5.0	25.8 ± 1.3	29.0 ± 5.3	*0.196*
NT-proBNP, pg/mL, median (IQR)	1126 (422–1919)	897 (191–1120)	1152 (495–2008)	*0.418*
Creatinine, mg/dL, median (IQR)	1.22 (0.98–1.44)	1.22 (1.22–1.34)	1.13 (0.97–1.45)	*0.517*
eGFR, ml/min/1.73m^2,^ mean ± SD	56.1 ± 24.2	53.4 ± 15.4	56.5 ± 25.4	*0.794*
NYHA stage, n (%)				*0.168*
*1*	7 (28.0%)	3 (60.0%)	4 (20.0%)	
*2*	14 (56.0%)	2 (40.0%)	12 (60.0%)	
*3*	4 (16.0%)	0 (0.0%)	4 (20.0%)	
**Medical History**				
Significant coronary artery disease, n (%)	7 (17.1%)	0 (0.0%)	7 (19.4%)	*0.279*
Myocardial infarction, n (%)	3 (7.3%)	0 (0.0%)	3 (8.3%)	*0.503*
Atrial fibrillation, n (%)	20 (48.8%)	2 (40.0%)	18 (50.0%)	*0.675*
Arterial Hypertension, n (%)	27 (65.9%)	2 (40.0%)	25 (69.4%)	*0.193*
Diabetes mellitus, n (%)	10 (25.0%)	1 (20.0%)	9 (25.7%)	*0.783*
Hyperlipidaemia, n (%)	18 (45.0%)	4 (80.0%)	14 (40.0%)	*0.093*
Chronic kidney disease, n (%)	24 (58.5%)	4 (80.0%)	20 (55.6%)	*0.299*
Valvular heart disease, n (%)	6 (14.6%)	0 (0.0%)	6 (16.7%)	*0.323*
Malignancy, n (%)	14 (34.1%)	0 (0.0%)	14 (38.9%)	*0.086*
**Concomitant therapy**				
ACE inhibitors/AT_1_ blocker, n (%)	25 (62.5%)	4 (80%)	11 (31.4%)	***0.036***
Beta blocker, n (%)	30 (75.0%)	3 (60.0%)	27 (77.1%)	*0.408*
Calcium channel blockers, n (%)	13 (32.5%)	0 (0.0%)	13 (37.1%)	*0.097*
Loop diuretics, n (%)	14 (35.0%)	1 (20.0%)	13 (37.1%)	*0.452*
Mineralocorticoid antagonists, n (%)	11 (27.5%)	1 (20.0%)	10 (28.6%)	*0.688*
SGLT-2 inhibitors, n (%)	9 (22.5%)	1 (20.0%)	8 (22.9%)	*0.886*

**Abbreviations.** ACE, angiotensin converting enzyme; AT_1_, angiotensin receptor 1; BMI, body-mass index; eGFR, estimated glomerular filtration rate; IQR, interquartile range; NT-proBNP, N-terminal prohormone of brain natriuretic peptide; NYHA, New York Heart Association stage; SD, standard deviation; SGLT-2, sodium-glucose cotransporter 2

*Categorical data is displayed as counts (percentage). Metric data are given as mean ± standard deviation in case of normal distribution*,* and as median (interquartile range) in case of non-normal distribution*,* which is assessed utilising the Shapiro-Wilk test.*

The majority (*n* = 32, 78.0%) of all scans were ordered for a suspicion of cardiac amyloidosis, while the rest (*n* = 9, 22.0%) were performed in the context of malignancy. All 41 [^99m^Tc]-DPD scans were visually graded as Perugini grade 1. The mean H/CL ratio was 1.15 ± 0.14. On subsequently acquired thoracic SPECT/CT images, true LV myocardial tracer uptake was confirmed in 4 (9.8% of total) patients, with 1 (2.4% of total) patient presented with focal uptake. All of these 5 (12.2% of total) patients had been referred to [^99m^Tc]-DPD scintigraphy and SPECT/CT imaging with a suspicion of cardiac amyloidosis. Genetic testing was performed 3 of these 5 patients, and returned positive in 1 patient. In all other patients (*n* = 36, 87.8% of total), LV tracer uptake was not myocardial but rather LV non-myocardial diffuse or blood-pool uptake.

### Myocardial vs. diffuse non-myocardial [^99m^Tc]-DPD tracer uptake

As described in the Table 2, H/CL ratio did not differ between patients with myocardial versus diffuse non-myocardial [^99m^Tc]-DPD uptake (1.21 ± 0.08 vs. 1.15 ± 0.14, *p* = 0.337), the LV myocardial uptake measured on the SPECT/CT images as SUV_peak_ significantly differed between groups [3.55 (IQR: 2.76–4.26) vs. 1.32 (IQR: 1.01–1.90), *p* = 0.010], also resulting in a significantly different tracer RI [1.46 (IQR: 0.84–2.67) vs. 0.34 (IQR: 0.17–0.46), *p* = 0.001]. Patients with LV myocardial uptake more frequently presented with history of carpal tunnel syndrome [3 (60.0%) vs. 4 (13.3%), *p* = 0.016] and apical sparing in echocardiography [2 (40.0%) vs. 0 (0.0%), *p* = 0.001]. Beyond that, concerning clinical and laboratory characteristics, no further significant differences between the two groups were observed.

**Table 2:** Imaging characteristics of the patient cohort

Variable	Total	Myocardial uptake	Diffuse uptake	*P*-value
	*N* = 41	*N* = 5	*N* = 36	
Indication				
Cardiac amyloidosis, n (%)	32 (78.0%)	5 (100.0%)	27 (75.0%)	
Malignancy, n (%)	9 (22.0%)	0 (0.0%)	9 (25.0%)	*0.206*
**Planar DPD-Scan**				
Heart-contralateral ratio, mean ± SD	1.15 ± 0.14	1.21 ± 0.08	1.15 ± 0.14	*0.337*
Perugini grade 1, n (%)	41 (100%)	5 (100.0%)	36 (100.0%)	
**DPD-SPECT/CT**				
Left ventricular uptake, n (%)	4 (9.8%)	4 (80.0%)	0 (0.0%)	
Focal LV myocardial uptake, n (%)	1 (2.4%)	1 (20.0%)	0 (0.0%)	
Diffuse non-myocardial uptake, (%)	36 (87.8%)	0 (0.0%)	36 (100.0%)	
SUV peak myocardium, median (IQR)	1.47 (1.03–2.19)	3.55 (2.76–4.26)	1.32 (1.01–1.90)	***0.010***
SUV peak vertebral, median (IQR)	7.44 (6.39–8.45)	6.47 (5.22–7.49)	7.50 (6.43–8.45)	*0.262*
SUV peak muscle, median (IQR)	1.82 (1.16–2.18)	2.27 (1.92–3.43)	1.80 (1.13–2.13)	*0.118*
SUV retention index, median (IQR)	0.37 (0.18–0.57)	1.46 (0.84–2.67)	0.34 (0.17–0.46)	***0.001***
**Cardiac magnetic resonance imaging**	*N* = 21*	*N* = 5	*N* = 16*	
LV ED diameter, mm, mean ± SD	46 ± 8	47 ± 9	46 ± 8	*0.921*
RV ED diameter, mm, mean ± SD	40 ± 8	39 ± 9	40 ± 8	*0.814*
Interventricular septum, mm, mean ± SD	14 ± 4	16 ± 3	14 ± 5	*0.475*
LV ED volume, ml, mean ± SD	158 ± 58	168 ± 79	155 ± 52	*0.665*
*LV ED volume index*,* ml/m*^*2*^, *mean ± SD*	*82 ± 29*	*88* ± 44	*80* ± 24	*0.630*
LV ejection fraction, %, mean ± SD	54 ± 14	53 ± 16	54 ± 14	*0.868*
LV CO, l/min, mean ± SD	5.2 ± 1.5	4.9 ± 1.2	5.3 ± 1.6	*0.542*
*LV CO index*,* l/min/m*^*2*^, *mean ± SD*	*2.7 ± 0.7*	*2.5 ± 0.6*	*2.8 ± 0.8*	*0.474*
RV ejection fraction, %, mean ± SD	55 ± 8	56 ± 8	54 ± 8	*0.760*
RV CO, l/min, mean ± SD	4.8 ± 1.3	4.3 ± 1.1	5.0 ± 1.3	*0.286*
*RV CO index*,* l/min/m*^*2*^, *mean ± SD*	*2.5 ± 0.7*	*2.2* ± 0.5	*2.6* ± 0.7	*0.238*
LV mass, g, mean ± SD	124 ± 40	142 ± 33	117 ± 41	*0.218*
T1 time, ms, median (IQR)	1045 (1011–1086)	1010 (988–1086)	1048 (1022–1082)	*0.405*
ECV, %, median (IQR)	28 (27–31)	28 (28–35)	28 (27–30)	*0.452*
Any LGE, n (%)	12 (60.0%)	4 (80.0%)	8 (53.3%)	*0.292*
*Amyloid-typical LGE*,* n (%)*	*1 (5.0%)*	*1 (20.0%)*	*0 (0.0%)*	*0.076*

**Abbreviations.** CO, cardiac output; ECV, extracellular volume; ED, end-diastolic; IQR, interquartile range; LGE, late gadolinium enhancement; LV, left ventricular; RV, right ventricular; SD, standard deviation; SUV, standardized uptake value

*Categorical data is displayed as counts (percentage). Metric data are given as mean ± standard deviation in case of normal distribution*,* and as median (interquartile range) in case of non-normal distribution*,* which is assessed utilising the Shapiro-Wilk test.*

* late gadolinium enhancement not available in 1 patient

In CMR imaging, out of five patients in whom myocardial LV uptake was confirmed, four demonstrated LGE. However, in only one patient, this was considered an amyloid-typical pattern. ECV did also not significantly differ between those with myocardial versus diffuse non-myocardial [^99m^Tc]-DPD uptake [28% (28–35) vs. 28% (27–30), *p* = 0.452]. All imaging parameters stratified by myocardial versus diffuse non-myocardial uptake are shown in Table 2.

### [^99m^Tc]-DPD SPECT/CT follow-up

Ten patients (24.4% of total) underwent nuclear medicine imaging follow-up by using [^99m^Tc]-DPD bone scintigraphy and thoracic SPECT/CT after a median of 26 (17–49) months. These included 7 patients who were previously found to have diffuse uptake, and 3 patients who previously presented with LV myocardial uptake. Clinical progression was observed in 3 patients (2 with LV myocardial uptake, and 1 with diffuse non-myocardial uptake). NYHA functional classes remained largely constant. However, one patient with LV myocardial uptake at baseline developed symptoms in line with polyneuropathy, and two patients (1 with LV myocardial and one with diffuse non-myocardial uptake) received a pacemaker within the follow-up period, one of these associated with a transaortic valve replacement procedure. With regard to medication prescription patterns, no significant changes were observed. NT-proBNP also remained largely constant. One patient with LV myocardial uptake demonstrated an increase in NT-proBNP (176 to 355 pg/mL) and one patient with diffuse non-myocardial uptake developed acute cardiac decompensation due to torrential tricuspid regurgitation.

**Fig. 3:**
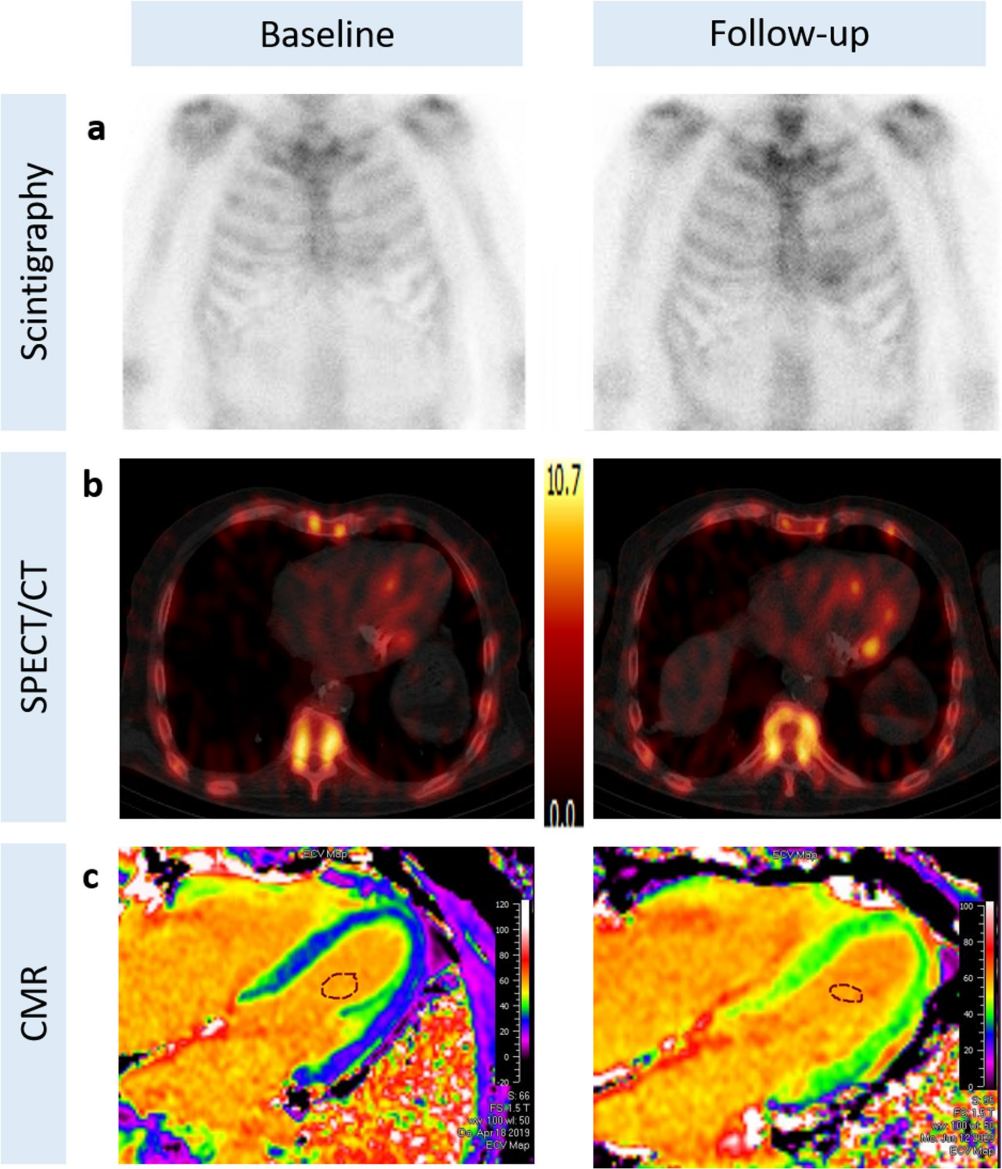
Representative nuclear medicine and CMR images showing the progression of ATTR-CM from baseline (left panels) to follow-up (right panels). Planar scintigraphic images (**a**) show the progression of the disease from Perugini grade 1 (baseline) to Perugini grade 2 (follow-up), confirmed also by SPECT/CT (**b**) and CMR ECV-maps (**c**). ATTR-CM, transthyretin amyloid cardiomyopathy; CMR, cardiac magnetic resonance; ECV, extracellular volume; SPECT/CT, single photon emission computed tomography/computed tomography

**Fig. 4:**
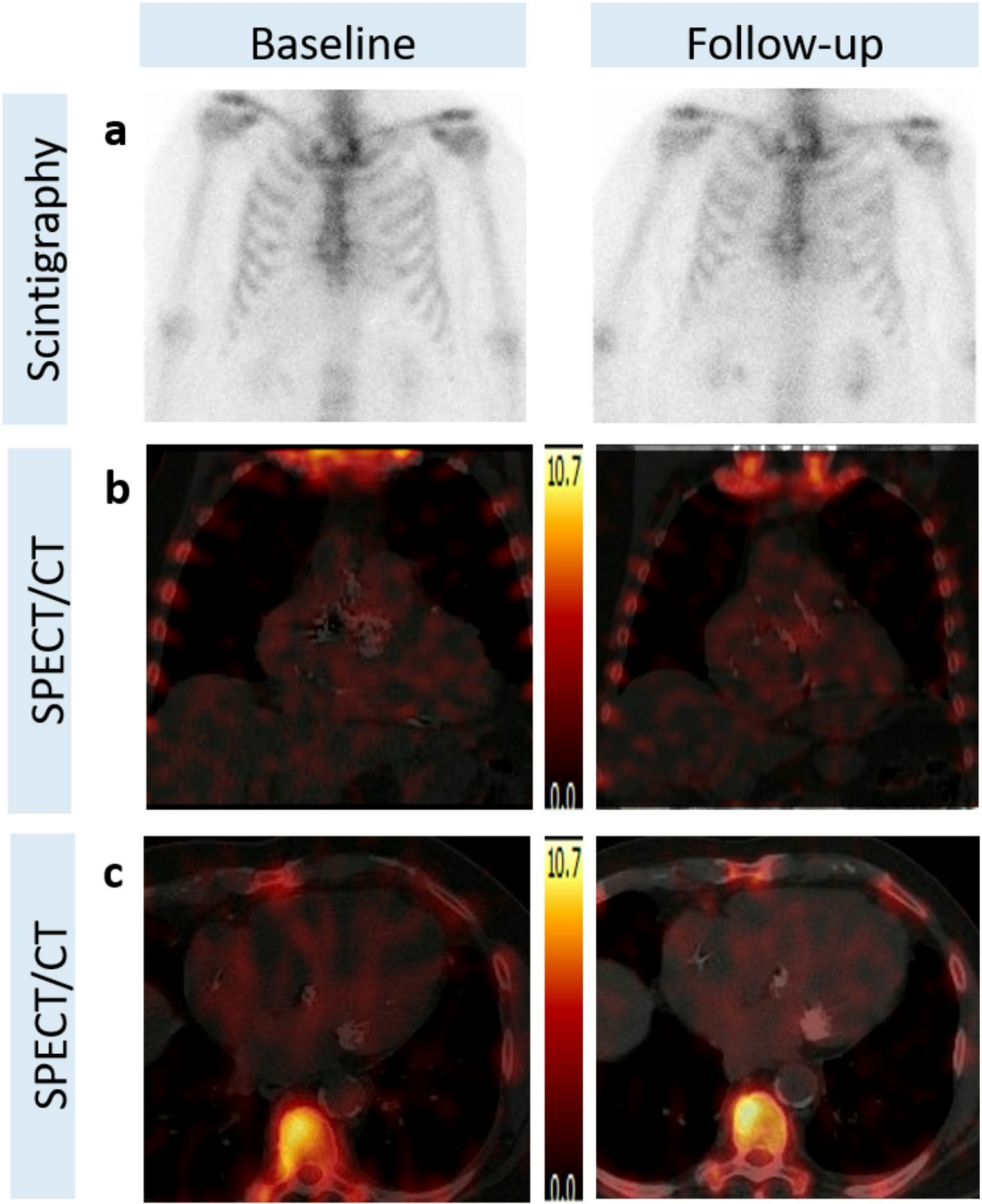
Representative scintigraphic images (**a**) of a patient with Perugini grade 1 at baseline (left) and at follow-up (right). SPECT/CT depicts the absence of LV uptake on the coronal (**b**) as well as on the transaxial (**c**) images in both examinations, baseline (left) and at follow-up (right), respectively

All patients who initially presented with LV myocardial uptake progressed to a Perugini grade 2 uptake and were concurrently diagnosed with ATTR-CM (Fig. 3) while among the 7 patients, who initially presented with diffuse uptake, diffuse non-myocardial uptake was confirmed in follow-up [^99m^Tc]-DPD-SPECT/CT (Fig. 4) in all but one patient, who demonstrated weak unspecific focal septal uptake.

Out of the remaining two patients who initially presented with myocardial uptake in SPECT/CT but did not complete a follow-up [^99m^Tc]-DPD-SPECT/CT examination, one was diagnosed with a variant ATTR gene shortly after the first scan, while the other was lost to follow-up.

## Discussion

In this retrospective study, we have demonstrated the relevant additional diagnostic value of performing thoracic SPECT/CT imaging in patients who underwent bone scintigraphy for a diagnosis of cardiac amyloidosis. The proportion of patients who demonstrated myocardial uptake (*n* = 5, 12.2%), as compared to diffuse non-myocardial or blood pool-associated uptake (*n* = 36, 87.8%), was very low. These patients, however, did eventually progress to Perugini grade 2 on follow-up, therefore enabling a non-invasive diagnosis of ATTR-CM.

While SPECT/CT imaging in addition to planar [^99m^Tc]-DPD scintigraphy is recommended in the most recent expert consensus statement on the diagnosis of cardiac amyloidosis [10], this is the first analysis to demonstrate the high proportion of patients with diffuse non-myocardial tracer uptake, which cannot be discerned from myocardial tracer uptake, if only planar imaging is utilised. Our findings, therefore, reinforce that SPECT/CT imaging should be routinely performed, especially in patients with scintigraphic evidence of Perugini grade 1, in addition to planar image acquisition.

Currently, the exact reasons for diffuse non-myocardial tracer uptake remain largely unknown. While several factors, which may cause false-positive scans have been described, e.g., recent myocardial infarction, chemotoxicity, annular calcification, or cardiac AL amyloidosis [10], we assume that blood pool uptake was the primary driver of false-positive results in our cohort. Naturally, the identification of factors which increase or lower the probability of a false-positive scan beyond classical risk factors for ATTR-CM (i.e., apical sparing pattern in echocardiography, history of spinal canal stenosis or carpal tunnel syndrome) would be of interest. However, owing to the relatively small sample size, we were also unable to attribute diffuse non-myocardial tracer uptake to any single factor specifically.

As various disease-modifying therapeutics, which can halt disease progression [14–16] become available, early recognition of ATTR-CM may be considered essential. With an increasing awareness for cardiac amyloidosis, it may be hypothesised that more patients will be referred to [^99m^Tc]-DPD scintigraphy in the future. This may well result in more frequent cases of Perugini grade 1 uptake. In these patients, SPECT/CT is crucial to assess and to confirm whether tracer uptake may truly be attributed to myocardial tissue – and therefore suggestive of ATTR-CM – or rather due to the blood pool.

While we suppose that Perugini grade 1 with confirmed myocardial tracer uptake in SPECT/CT is indicative of ATTR-CM in patients with unremarkable IFIX/FLC assessment, the non-invasive diagnostic algorithm proposed by Gillmore et al. [6] was evaluated for Perugini grades 2 and 3, but not grade 1. Indeed, Verheyen et al. [17] have revisited the diagnostic accuracy of this non-invasive diagnostic algorithm [6] using histopathological samples of patients who have undergone both bone scintigraphy and EMB. In their analysis, bone scintigraphy with mild or absent cardiac tracer uptake neither confirmed nor excluded cardiac amyloidosis, even in patients with no detectable monoclonal paraprotein and an unremarkable FLC assessment. Therefore, the authors recommend tissue biopsy in any other constellation than Perugini grade ≥ 2 uptake in bone scintigraphy and negative FLC. In our view, this supports the retained diagnostic value of CMR and, if in doubt, EMB, especially in patients who present with clinical deterioration.

Conversely, in a recent multi-centre analysis of patients with asymptomatic ATTR-CM [18], the authors also considered [^99m^Tc]-DPD scintigraphy with Perugini grade 1 and confirmed LV myocardial tracer uptake in SPECT/CT indicative of ATTR-CM, provided a negative IFIX/FLC assessment. Compared to asymptomatic patients with more advanced Perugini stages, these patients had a remarkably better outcome, with the median survival being significantly longer than in patients presenting with Perugini grades 2 or 3 and a lower proportion of cardiac events [18]. This highlights the apparently already adopted practice of extending the non-invasive diagnostic algorithm proposed by Gillmore et al. [6] to interpret Perugini grade 1 with confirmed myocardial tracer uptake in SPECT/CT as indicative of ATTR-CM, provided a negative IFIX/FLC assessment.

While the role for [^99m^Tc]-DPD scintigraphy and SPECT/CT is considered of value primarily for diagnostic purposes, it may also be suitable to evaluate disease severity and progression. For instance, an association of the degree of LV myocardial tracer uptake with echo-based markers of LV function [12] and even ECV in CMR [19] has previously been demonstrated. The degree of myocardial tracer uptake quantified utilising the SUV has been linked to exercise capacity in cardiopulmonary exercise testing [20]. Furthermore, [^99m^Tc]-DPD scintigraphy featuring SPECT/CT imaging may also be suitable to monitor treatment response in disease-specific ATTR-CM therapeutics [21, 22]. Finally, this imaging modality may even have the capacity to evaluate the possibility of right ventricular involvement in ATTR-CM, which is also considered to be associated with adverse outcomes [23]. This cumulative evidence suggests that the value of [^99m^Tc]-DPD scintigraphy and thoracic SPECT/CT may go beyond a tool for primary diagnosis but could also be utilised throughout the ATTR-CM disease spectrum, from very early disease, as in our case, towards more advanced disease stages.

In our albeit small patient cohort, CMR was unable to reliably differentiate between patients with LV tissular myocardial [^99m^Tc]-DPD uptake versus diffuse non-myocardial tracer uptake. Therefore, we speculate that [^99m^Tc]-DPD scintigraphy featuring thoracic SPECT/CT imaging may indeed be more sensitive in very early ATTR-CM disease stages. However, the immense clinical value of CMR, especially featuring gadolinium-enhanced imaging and parametric mapping techniques, lies in its broad applicability for establishing differential diagnoses, including light chain amyloidosis, and other causes of a hypertrophic cardiac phenotype. In practice, CMR is also likely to be more readily available, less time-consuming, and also free of radiation. In case of discrepant findings in other diagnostic imaging modalities, it might be sensible to combine modalities to establish a diagnosis in these patients, especially to exclude differential diagnosis, which could potentially also result in very mild [^99m^Tc]-DPD tracer uptake, including pericarditis, recent myocardial infarction, or rib fractures [10].

In clinical practice, patients with Perugini grade 1 tracer uptake pose a diagnostic challenge to the treating physicians due to a lack of evidence and best practices in these patients. This is further complicated as clinically, these patients seem to hardly differ from other patients with various aetiologies of HF or chronic kidney disease. While there is currently no consensus and minimal data on the treatment of very early ATTR-CM, we assume that repeated [^99m^Tc]-DPD scintigraphy may be a suitable tool to detect potential disease progression in Perugini grade 1 patients with proven myocardial uptake on SPECT/CT. However, as this assumption is based on a very small number of patients, EMB ought to remain the gold standard for the diagnosis of cardiac amyloidosis, if in doubt.

However, as Porcari et al. [18] have recently demonstrated, the course of disease in Perugini grade 1 patients seems to be relatively benign. These clinical findings are in line with another imaging study, which analysed SUVs as a predictor of outcome [12]. Therefore, repeated [^99m^Tc]-DPD scintigraphy and SPECT/CT for the detection of disease progression may also be a feasible option in selected patients who opt not to undergo EMB and present with unremarkable findings in CMR. Currently, there is no evidence on the specific interval which should be applied for repeated diagnostic work-up. However, as previous studies which assessed temporal changes in [^99m^Tc]-DPD uptake have described median follow-up periods of 9 to 28 months [21, 24], we suggest that diagnostic work-up may be repeated after 18 to 24 months if no clinical progression is observed. Importantly, in our view, repeated work-up needs to additionally include IFIX/FLC and CMR for differential diagnosis. In case of clinical progression, patients should undergo EMB or, if unfeasible, repeated non-invasive assessment at a tertiary referral centre.

Importantly, bone scintigraphies and subsequent SPECT/CT imaging were performed in line with our centre’s standard operating procedure and used exclusively [^99m^Tc]-DPD. Therefore, this analysis cannot be utilised to draw any conclusion concerning other commercially available tracer substances like pyrophosphate- or hydroxymethylene diphosphonate (HMDP)-based tracers. While the evaluation of other tracer substances was not the aim of our current analysis, the literature suggests that there are indeed some differences in the respective tracers’ affinity for transthyretin amyloid, at least between [^99m^Tc]-DPD and [^99m^Tc]-HMDP [25].

### Limitations

Due to the retrospective nature of this analysis, certain limitations inherent to this study design need to be considered. First, as a tertiary referral centre for cardiac amyloidosis and nuclear medicine, certain biases regarding patient selection cannot be entirely excluded. Especially the referral rate and proportion of indications for [^99m^Tc]-DPD scintigraphy (amyloid vs. malignancy) may not be representative for other patient cohorts. Especially in oncologic patients, cardiac tracer uptake was only described as an incidental finding on planar bone scans, and SPECT/CT was therefore not performed in all cases, which lead to the exclusion of several patients from this analysis. Finally, relatively few patients completed follow-up in our cohort.

### Future research questions

As our analysis was limited to a single-centre cohort, our findings ought to be confirmed in larger and ideally multi-centre cohorts. Furthermore, more data is needed on whether Perugini grade 1 patients with myocardial tracer uptake eventually progress to Perugini grade 2, which would reinforce the notion that these patients should be suspected to have, or even be diagnosed with, very early ATTR-CM.

## Conclusion

Additional thoracic SPECT/CT is highly advisable in all patients with suspected cardiac amyloidosis who demonstrate mild cardiac tracer uptake, scored as Perugini grade 1. The rate of non-myocardial tracer uptake seems to be relatively high, with well above 80% in our cohort. Among patients with mild LV myocardial [^99m^Tc]-DPD tracer uptake confirmed in SPECT/CT imaging, progression to Perugini grade 2 was observed in all patients who underwent follow-up, though this only applied for three patients. Concurrently, these patients were diagnosed with ATTR-CM non-invasively.

## Data Availability

The datasets generated during and/or analyzed during the current study are available from the corresponding author on reasonable request.

## References

[1] Wechalekar AD, Gillmore JD, Hawkins PN. Systemic amyloidosis. Lancet [Internet]. Lancet; 2016 [cited 2022 Jul 29];387:2641–54. 10.1016/S0140-6736(15)01274-X 10.1016/S0140-6736(15)01274-X 26719234

[2] Grogan M, Scott CG, Kyle RA, Zeldenrust SR, Gertz MA, Lin G, et al. Natural history of wild-type transthyretin cardiac amyloidosis and risk stratification using a novel staging system. J Am Coll Cardiol. 2016;68:1014–20. 10.1016/J.JACC.2016.06.033. 27585505 10.1016/j.jacc.2016.06.033

[3] Damy T, Costes B, Hagège AA, Donal E, Eicher JC, Slama M, et al. Prevalence and clinical phenotype of hereditary transthyretin amyloid cardiomyopathy in patients with increased left ventricular wall thickness. Eur Heart J. 2016;37:1826–34. 10.1093/EURHEARTJ/EHV583. 26537620 10.1093/eurheartj/ehv583

[4] Rozenbaum MH, Large S, Bhambri R, Stewart M, Whelan J, van Doornewaard A, et al. Impact of delayed diagnosis and misdiagnosis for patients with transthyretin amyloid cardiomyopathy (ATTR-CM): a targeted literature review. Cardiol Ther. 2021;10:141–59. 10.1007/S40119-021-00219-5. 33877591 10.1007/s40119-021-00219-5 PMC8126532

[5] Damy T, Adams D, Bridoux F, Grateau G, Planté-Bordeneuve V, Ghiron Y, et al. Amyloidosis from the patient perspective: the French daily impact of amyloidosis study. Amyloid. 2022;29(3):165–74. 10.1080/13506129.2022.2035354. 35144512 10.1080/13506129.2022.2035354

[6] Gillmore JD, Maurer MS, Falk RH, Merlini G, Damy T, Dispenzieri A, et al. Nonbiopsy diagnosis of cardiac transthyretin amyloidosis. Circulation. 2016;133:2404–12. 10.1161/CIRCULATIONAHA.116.021612. 27143678 10.1161/CIRCULATIONAHA.116.021612

[7] Perugini E, Guidalotti PL, Salvi F, Cooke RMT, Pettinato C, Riva L, et al. Noninvasive etiologic diagnosis of cardiac amyloidosis using 99mTc-3,3-diphosphono-1,2-propanodicarboxylic acid scintigraphy. J Am Coll Cardiol. 2005;46:1076–84. 10.1016/J.JACC.2005.05.073. 16168294 10.1016/j.jacc.2005.05.073

[8] Writing Committee, Kittleson MM, Ruberg FL, Ambardekar A, Brannagan TH, Cheng RK, et al. 2023 ACC expert consensus decision pathway on comprehensive multidisciplinary care for the patient with cardiac amyloidosis. J Am Coll Cardiol. 2023. 10.1016/J.JACC.2022.11.022. 10.1016/j.jacc.2022.11.022 36697326

[9] Ruan D, Sun L. Diagnostic efficacy of bone scintigraphy in transthyretin cardiac amyloidosis: an updated systematic review and bayesian bivariate meta-analysis. Clin Transl Imaging [Internet]. Springer Science and Business Media Deutschland GmbH; 2022 [cited 2023 Feb 10];10:85–98. 10.1007/S40336-021-00471-8/FIGURES/6

[10] Garcia-Pavia P, Rapezzi C, Adler Y, Arad M, Basso C, Brucato A, et al. Diagnosis and treatment of cardiac amyloidosis: a position statement of the ESC working group on myocardial and pericardial diseases. Eur Heart J. 2021;42:1554–68. 10.1093/EURHEARTJ/EHAB072. 33825853 10.1093/eurheartj/ehab072 PMC8060056

[11] Castaño A, DeLuca A, Weinberg R, Pozniakoff T, Blaner WS, Pirmohamed A, et al. Serial scanning with technetium pyrophosphate (99mTc-PYP) in advanced ATTR cardiac amyloidosis. J Nucl Cardiol. 2016;23:1355–63. 10.1007/s12350-015-0261-x. 10.1007/s12350-015-0261-x PMC4826633 26453570

[12] Rettl R, Duca F, Kronberger C, Binder C, Willixhofer R, Ermolaev N, et al. Prognostic implication of DPD quantification in transthyretin cardiac amyloidosis. Eur Heart J. 2024;(2):260. 10.1093/EHJCI/JEAE295. 10.1093/ehjci/jeae295 PMC11781830 39545930

[13] Kammerlander AA, Donà C, Nitsche C, Koschutnik M, Schönbauer R, Duca F, et al. Feature tracking of global longitudinal strain by using cardiovascular MRI improves risk stratification in heart failure with preserved ejection fraction. Radiology. 2020;296(2):290–8. 10.1148/RADIOL.2020200195. 32484413 10.1148/radiol.2020200195

[14] Maurer MS, Schwartz JH, Gundapaneni B, Elliott PM, Merlini G, Waddington-Cruz M, et al. Tafamidis treatment for patients with transthyretin amyloid cardiomyopathy. N Engl J Med. 2018;379:1007–16. 10.1056/NEJMOA1805689. 30145929 10.1056/NEJMoa1805689

[15] Gillmore JD, Judge DP, Cappelli F, Fontana M, Garcia-Pavia P, Gibbs S, et al. Efficacy and safety of acoramidis in transthyretin amyloid cardiomyopathy. N Engl J Med. 2024;390(2):132–42. 10.1056/NEJMOA2305434. 38197816 10.1056/NEJMoa2305434

[16] Fontana M, Berk JL, Gillmore JD, Witteles RM, Grogan M, Drachman B, et al. Vutrisiran in patients with transthyretin amyloidosis with cardiomyopathy. N Engl J Med. 2025. 10.1056/NEJMOA2409134. 39213194 10.1056/NEJMoa2409134

[17] Verheyen N, Ungericht M, Paar L, Danninger K, Schneiderbauer-Porod S, Duca F, et al. Diagnostic accuracy of bone scintigraphy for the histopathological diagnosis of cardiac transthyretin amyloidosis—a retrospective Austrian multicenter study. Biomedicines. 2022. 10.3390/BIOMEDICINES10123052. 10.3390/biomedicines10123052 PMC9775679 36551808

[18] Porcari A, Razvi Y, Cappelli F, Nitsche C, Serenelli M, Longhi S, et al. Clinical phenotype and prognosis of asymptomatic patients with transthyretin cardiac amyloid infiltration. JAMA Cardiol. 2025;10(5):437. 10.1001/JAMACARDIO.2024.5221. 39841451 10.1001/jamacardio.2024.5221 PMC12079285

[19] Rettl R, Calabretta R, Duca F, Kronberger C, Binder C, Willixhofer R, et al. DPD quantification correlates with extracellular volume and disease severity in wild-type transthyretin cardiac amyloidosis. JACC: Adv. [Internet]. Elsevier B.V.; 2024 [cited 2025 May 16];3. 10.1016/j.jacadv.2024.101261. 10.1016/j.jacadv.2024.101261 PMC11416666 39309666

[20] Ermolaev N, Rettl R, Willixhofer R, Kronberger C, Poledniczek M, Schmid LM, et al. Cardiopulmonary exercise testing correlates with quantitative left ventricular [99mTc]-DPD uptake in transthyretin amyloid cardiomyopathy. J Clin Med. 2025;14:2999. 10.3390/JCM14092999. 40364030 10.3390/jcm14092999 PMC12072802

[21] Rettl R, Wollenweber T, Duca F, Binder C, Cherouny B, Dachs TM, et al. Monitoring tafamidis treatment with quantitative SPECT/CT in transthyretin amyloid cardiomyopathy. Eur Heart J. 2023;24:1019–30. 10.1093/EHJCI/JEAD030. 10.1093/ehjci/jead030 PMC10364619 36881774

[22] Rettl R, Calabretta R, Duca F, Binder C, Kronberger C, Willixhofer R, et al. Reduction in 99mTc-DPD myocardial uptake with therapy of ATTR cardiomyopathy. Amyloid. 2024;31(1):42–51. 10.1080/13506129.2023.2247136. 10.1080/13506129.2023.2247136 37599395

[23] Zhao M, Calabretta R, Binder P, Yu J, Jiang Z, Nitsche C, et al. Clinical significance of quantitative assessment of right ventricular amyloid burden with [99mTc]Tc-DPD SPECT/CT in transthyretin cardiac amyloidosis. Eur J Nucl Med Mol Imaging. 2024. 10.1007/S00259-024-06981-7. 10.1007/s00259-024-06981-7 39586845

[24] Razvi Y, Porcari A, Hutt DF, Lazari J, Ioannou A, Patel RK, et al. Uncertain clinical relevance of serial bone scintigraphy findings in treated transthyretin amyloid cardiomyopathy. JACC Cardiovasc Imaging. 2025;18(8):899–908. 10.1016/j.jcmg.2025.03.014. 10.1016/j.jcmg.2025.03.014 40637650

[25] Porcari A, Hutt DF, Grigore SF, Quigley AM, Rowczenio D, Gilbertson J et al. Comparison of different technetium-99m-labelled bone tracers for imaging cardiac amyloidosis. Eur J Prev Cardiol [Internet]. Oxford Academic; 2023 [cited 2025 Sep 23];30:e4–6. 10.1093/EURJPC/ZWAC237 10.1093/eurjpc/zwac237 36256685

